# A Simple Bedside Exercise Method to Enhance Lower Limb Muscle Strength in Moderate Alzheimer’s Disease Patients with Sarcopenia

**DOI:** 10.3390/healthcare9060680

**Published:** 2021-06-04

**Authors:** Jung Hae Yun, Du Hwan Kim, Min Cheol Chang

**Affiliations:** 1 Department of Physical Medicine and Rehabilitation, College of Medicine, Yeungnam University, Daegu 705-717, Korea; junghae5660@gmail.com; 2 Department of Physical Medicine and Rehabilitation, College of Medicine, Chung-Ang University, Seoul 06973, Korea; ri-pheonix@hanmail.net

**Keywords:** muscle strength, muscle mass, ageing

## Abstract

Background: Alzheimer’s disease (AD) is known to accelerate muscle loss in the elderly due to reduced physical performance, increasing the prevalence and severity of sarcopenia. This study was undertaken to determine whether simple bedside exercise training may facilitate muscle growth and strengthening in moderate-degree AD patients. Methods: This study was conducted on 26 prospectively recruited women admitted to a nursing hospital, who had moderate AD and sarcopenia. They were randomly and evenly divided into the control and exercise groups. For five sessions per week, those in the exercise group underwent 30 min of therapist-supervised exercise by simply kicking a balloon connected to the ceiling by a piece of string while lying on a bed. Additional exercise was encouraged, and isometric maximal voluntary contraction (MVC) and skeletal muscle mass index (SMI) were measured and calculated after 12 weeks. Results: Through simple exercise training for 12 weeks, MVCs for hip flexion and knee extension significantly increased in the exercise group. However, no significant differences in SMI were found between the two groups. Conclusions: We believe that our simple exercise method can be applied to patients with AD for maintaining and enhancing the strength of the muscles of the lower extremities.

## 1. Introduction

Alzheimer’s disease (AD) is one of the most prevalent neurodegenerative diseases in the elderly, characterized by its irreversibility and progressive functional, cognitive, and behavioral loss [1]. As the average life expectancy of worldwide population increases continuously, the prevalence of AD rises as well. Many studies have demonstrated demographic risk factors for AD, including genetic, lifestyle, and sex-related characteristics. In terms of sex-related characteristics, AD has been known to be more prevalent in women [1].

AD is one of the leading concomitant diseases that increase the risk of sarcopenia, which is defined as the loss of skeletal muscle mass and low muscle strength with or without poor physical performance with aging. Sarcopenia has been described as a comorbidity that accompanies immobility [2]. AD increases the risk of sarcopenia due to a worsened physical performance. Decreased physical performance, such as gait disturbance or declined activities of daily living, is thought to be correlated with cognitive dysfunction in AD, leading to a higher risk of sarcopenia [3]. A previous study demonstrated the relationship between muscle strength decline and hippocampal atrophy in AD [4]. To prevent progression of muscle loss and strength, exercise training in everyday life is important [5]. However, AD patients usually show poor exercise compliance due to their impaired cognitive function and physical activity. AD patients also spend a lot of time in bed due to poor ambulatory function. Therefore, it is necessary to develop a simple and easy-to-do exercise program for patients with AD. This study sought to investigate the effectiveness of a simple exercise program on muscle function with AD, using a balloon-kicking program.

## 2. Methods

### 2.1. Subjects

This study prospectively recruited 26 women with moderate AD and sarcopenia aged 67 to 84 years (mean age, 78.2 ± 4.9 years) who were admitted to a nursing hospital between January 2019 and March 2019 (Table 1). Sarcopenia was defined as SMI < 5.7 kg/m^2^ by bioelectrical impedance analysis (BIA). The inclusion criteria were as follows: (1) older women with sarcopenia between 65 and 85 years of age; (2) the presence of moderate AD (Clinical Dementia Rating score: 2); (3) Modified Mini-Mental State Examination (MMSE; score 0–30) scores between 13 and 20; (4) able to obey an instruction to perform a certain simple movement; (5) no history of central nervous system diseases, such as stroke, traumatic brain injury, or myelopathy, other than AD; (6) the absence of serious medical complications, such as pneumonia or cardiac problems. Based on the previous study [6], the present study calculated a sample size based on isometric MVC. In that study, the difference of isometric MVC of hip flexor was 0.31 ± 0.24 (mean ± standard deviation). When we adopted type I error of 0.05, power of 80%, and two-sided test, 11 subjects per group were found to be necessary for our study. Considering 20% as the dropout rate, we needed to recruit 13 subjects. Those with MMSE scores above 20 and below 13 were excluded due to the following reasons. Patients with mild Alzheimer were excluded because they would benefit from a more active exercise due to their preserved ability to perform outdoor physical activity. Moderate and severe patients with an MMSE score below 12 were excluded as they presented difficulties in learning and exercising, regardless of the difficulty of the exercise program.

This study was conducted in compliance with the Code of Ethics of the World Medical Association (Declaration of Helsinki) for experiments involving humans. Our study protocol was approved by the institutional review board of Yeungnam University hospital. Written informed contents were obtained from the patients’ guardians.

### 2.2. Study Design

This study was designed and performed prospectively. All patients were randomly assigned to two groups: the exercise and control groups (*n* = 13/group). Randomization was performed via a random number table. Under the supervision of a physical therapist, each patient in the exercise group underwent five training sessions on consecutive days (Monday to Friday) every week for 12 weeks and received a total of 60 sessions.

### 2.3. Exercise (Kicking a Ballon)

**Figure 1:**
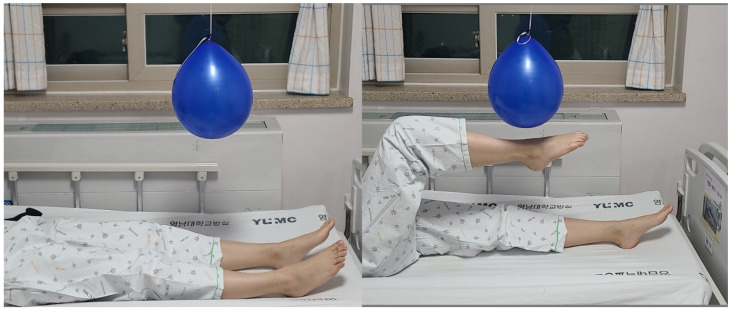
Balloon-kicking exercise was performed.

For the kicking exercise, this study connected a balloon to the ceiling using a piece of string. The inferior tip of the balloon was located approximately 60 cm above the level of a bed, above the distal one-third point of the patient’s tibia (Figure 1). Each patient performed the exercise in a supine position, kicking the balloon by extending the leg followed by hip flexion for 30 min, under supervision of a physical therapist to encourage and ensure that exercise was performed full time by following the appropriate method. The right and left extremities were evenly exercised for a similar duration. Patients were encouraged by nurses and caregivers to exercise as frequently as possible outside of training sessions and on weekends.

### 2.4. Muscle Strength Evaluation

Muscle strength was assessed by an examiner blinded to group allocation using a handheld dynamometer (MicroFet2; Hoggan Health Industries Inc.; West Jordan, UT, USA). Isometric MVC was assessed for bilateral hip flexion and knee extension. Three trials for each unilateral muscle group were performed with a 5 min rest between trials. The highest peak value among six trials was determined as the MVC for each muscle group. All MVC values were normalized to the total body weight (MVC/TBW).

### 2.5. Body Composition

Bioelectrical impedance analysis (BIA) was performed on all of the patients a day before and after the exercise sessions using the Inbody system (Inbody 720, Biospace, Seoul, South Korea) with an operating frequency of 50 kHz at 800 μA. It was conducted by an examiner blinded to group allocation. This instrument used eight tactile electrodes, with four placed on the palms and thumbs of both hands and the other four on the anterior and posterior parts of the soles of both feet. The patient stood with the soles touching the foot electrodes while grabbing the hand electrodes. The electrodes then send alternating low- and high-frequency electrical currents to calculate electrical resistance of one’s body composition. The skeletal muscle mass was calculated using the BIA equation as follows: skeletal muscle mass (kg) = (0.401 × [height^2^/resistance] + [3.825 × sex] − [0.071 × age] + 5.201]), where the height was measured in centimeters; resistance in ohms; for sex, men = 1 and women = 0, and age in years [6]. The SMI was calculated by dividing the skeletal muscle mass (kg) by the height squared (m^2^) [6].

### 2.6. Statistical Analysis

Data were analyzed using the Statistical Package for Social Sciences (SPSS) version 23.0 (IBM Corp., Armonk, NY, USA). The normality test was performed using the Kolmogorov–Smirnov test. The Mann–Whitney U test was used to evaluate the differences of the demographic data, muscle strength, and SMI prior to the exercise sessions between the exercise and control groups. The Wilcoxon signed rank test was used for the intragroup comparisons between data before and after the exercise sessions. To evaluate the effect of exercise, the difference in MVC and SMI between before and after exercise was calculated, and the comparison between both groups was performed using the Mann–Whitney U test. The level of statistical significance was set at *p* < 0.05.

## 3. Results

One patient in each group had dropped out due to worsening of general condition. No significant intergroup differences were observed in the demographic data (Table 1). Prior to initiating the exercise sessions, hip flexion MVC (exercise group: 0.98 ± 0.19, control group: 1.03 ± 0.22, *p* = 0.443) and knee extension MVC (exercise group: 1.26 ± 0.24, control group: 1.30 ± 0.17, *p* = 0.630) were not significantly different between both groups. After the sessions, in the exercise group, MVCs for hip flexion and knee extension were significantly increased (hip flexor: difference = 0.28 ± 0.15, *p =* 0.002; knee extensor: difference = 0.18 ± 0.07, *p* = 0.002). In contrast, in the control group, after 12 weeks, MVCs for hip flexion and knee extension were not significantly changed (hip flexor: difference = −0.01 ± 0.02, *p =* 0.122; knee extensor: difference = −0.02 ± 0.04, *p =* 0.124) (Table 2). The differences in MVCs for hip flexion and knee extension before and after the sessions were significantly larger in the exercise group, compared with the control group (*p* < 0.001). However, no significant differences in SMI after 12 weeks of sessions in exercise group and control group (exercise group: difference = 0.01 ± 0.07, *p =* 0.479; control group: difference = −0.01 ± 0.02, *p =* 0.590) were found, and no difference in SMI was noted after the sessions between the exercise and control groups (*p* = 0.160) (Table 2).

## 4. Discussion

A simple and easy-to-do exercise program is necessary for patients with AD. The present study sought to investigate the effectiveness of a simple exercise program, i.e., kicking a balloon connected to the ceiling while lying on a bed. All patients enrolled in the study underwent routine treatment, including nutritional support. The patients in the exercise group performed a three-month exercise program. After concluding the program, the patients’ MVCs of hip flexors and knee extensors were significantly increased compared with the exercise group baseline and the control group.

Due to a lack of physical activities, patients with AD are likely to develop sarcopenia [3]. Regular exercise is essential for preventing and treating sarcopenia. Therefore, we developed a simple exercise (kicking a balloon) that can be performed with sufficient compliance even if cognitive function is significantly reduced. We think that this exercise is advantageous in that it can be easily executed, even by the elderly with cognitive impairment. Additionally, compared with previous studies mainly performed as group-based exercise programs, special tools other than a balloon are not necessary [7,8]. In particular, considering the fact that AD patients spend most of their time in bed and have difficulty ambulating, kicking a balloon while in a lying position is an appropriate and convenient exercise for them.

Previous studies showed controversial results regarding whether exercise training increases muscles strength and mass in elderly patients with sarcopenia [9,10,11,12,13]. Though controversy remains, despite being a relatively simple and monotonous exercise, MVCs for hip flexion and knee extension were significantly increased after 3 months of exercise in our study. The strength of the hip flexors and knee extensors is a critical factor for human ambulation. However, our exercise did not increase muscle mass. This result may be due to the fact that no resistance was applied to the muscle group during the exercise; resistance exercise is known to facilitate muscle growth factors release, leading to muscle regeneration [14,15,16,17,18]. The study period of 12 weeks of exercise may also have been an inadequate duration to build up muscle mass [19]. Additionally, we conducted the exercise only on the lower extremities, which would be not enough to manifest as an increase in muscle mass throughout the whole body.

Similarly to our study, Thomas et al. evaluated the effect of a brief exercise training with AD patients [20]. This study prospectively recruited 28 patients with mild-to-moderate AD (mean MMSE score: 17.8 ± 7.2), and performed resistance training using Theraband (Akron, OH, USA) on the lower extremities. The exercise consisted of moderate-intensity progressive resistance training of the hip extensors, abductors, knee extensors and flexors, and ankle dorsiflexors using Theraband for 3 days weekly over six-week period. After the six-week exercise period, they found that there was an increase in muscle strength of quadriceps muscles, but the strength of iliopsoas muscle was reduced. The weakened iliopsoas muscle may be due to either poor compliance of AD patients in measuring muscle strength or lack of exercise time, demonstrating the importance of compliance and training time.

## 5. Conclusions

In conclusion, a well-designed and appropriate simple bedside exercise training for moderate-degree AD patients was performed. Our research is meaningful in that although it is a simple and monotonous exercise, it can be helpful to increase the muscle strength. However, this study is limited in that we recruited a relatively small number of subjects, and long-term follow-up was not conducted. In addition, the compliance of each patient was not objectively measured; however, certain participations were observed to be related to better outcomes. Further studies compensating these limitations, such as measuring emotional and functional participation of AD patients, are warranted in the future [21,22].

## Figures and Tables

**Table 1:** Patient demographics and baseline clinical data.

	Exercise Group (*n* = 13)	Control Group (*n* = 13)	Z Statics	*p*
Age (y)	78.3 ± 5.3	78.2 ± 4.8	−0.262	0.799
MMSE	18.8 ± 1.0	18.98 ± 1.0	−0.211	0.843
Weight (kg)	49.8 ± 3.4	49.7 ± 2.1	−0.232	0.843
Height (m)	1.53 ± 0.04	1.53 ± 0.03	−0.292	0.799
BMI (kg/m^2^)	21.4 ± 0.9	21.4 ± 0.6	−0.347	0.755
Baseline SMI (kg/ m^2^)	5.15 ± 0.30	5.16 ± 0.15	−0.145	0.887
MVC: hip flexor (N/kg)	0.98 ± 0.19	1.03 ± 0.22	−0.811	0.443
MVC: knee extensor (N/kg)	1.26 ± 0.24	1.30 ± 0.17	−0.491	0.630

Values are presented as mean ± standard deviation. MMSE: Mini-Mental State Examination; BMI: body mass index; SMI: skeletal muscle mass index; MVC: maximum voluntary contraction. *p*-value was calculated using the Mann–Whitney U test.

**Table 2:** Intragroup and intergroup differences of clinical data before and after the exercise sessions.

Variable	Group	Pre	Post	Difference	Intragroup		Intergroup	
					Z Statics	*p*	Z Statics	*p*
SMI	Exercise (*n* = 12)	5.15 ± 0.30	5.16 ± 0.28	0.01 ± 0.07	−0.708	0.479	−1.449	0.160
	Control (*n* = 12)	5.16 ± 0.15	5.15 ± 0.14	−0.01 ± 0.02	−1.887	0.590		
MVC: hip flexor (N/kg)	Exercise (*n* = 12)	0.98 ± 0.19	1.26 ± 0.24	0.28 ± 0.15	−3.062	**0.002**	−4.170	**<0.001**
	Control (*n* = 12)	1.03 ± 0.22	1.02 ± 0.21	−0.01 ± 0.02	−1.545	0.122		
MVC: knee extensor (N/kg)	Exercise (*n* = 12)	1.26 ± 0.24	1.45 ± 0.19	0.18 ± 0.07	−3.063	**0.002**	−4.165	**<0.001**
	Control (*n* = 12)	1.30 ± 0.17	1.27 ± 0.16	−0.02 ± 0.04	−1.539	0.124		

Values are presented as mean ± standard deviation. SMI: skeletal muscle mass index; MVC: maximum voluntary contraction. *P* value was calculated using the Mann–Whitney U test. Bold numbers are significant at *p* < 0.05.

## Data Availability

The data can be provided upon reasonable request to the corresponding author.
